# Geographic Distance and Time to Definitive Surgery for Primary Cutaneous Melanoma in the Toowoomba Regional Catchment: A Retrospective Audit

**DOI:** 10.7759/cureus.114196

**Published:** 2026-08-09

**Authors:** San Jack Low, Damian Fry, Patrick C Litchfield, Kelsey Reinheimer, Chinthani Madigasekara

**Affiliations:** 1 Surgery, Toowoomba Hospital, Toowoomba, AUS; 2 General Surgery, Darling Downs Hospital and Health Service, Toowoomba, AUS; 3 General Surgery, Toowoomba Base Hospital, Toowoomba, AUS; 4 General Surgery, Logan Hospital, Meadowbrook, AUS

**Keywords:** access to care, geographic distance, melanoma, rural health, sentinel lymph node biopsy, surgical delay

## Abstract

Background

Timely melanoma treatment is essential to minimise disease progression. Current guidelines recommend completion of diagnostic assessment, specialist referral, staging and definitive surgery within six weeks of biopsy. Toowoomba Hospital serves a large, geographically dispersed regional catchment, and travel distance may affect access to specialist melanoma care. This study examined the association between residential distance from Toowoomba and time from melanoma biopsy to definitive surgery.

Methods

A retrospective audit included patients who underwent wide local excision with sentinel lymph node biopsy for primary cutaneous melanoma at Toowoomba Hospital between 2021 and 2025. Time to surgery and the proportion treated within 42 days were compared across residential distance groups. Simple linear regression assessed the association between continuous distance and surgical interval.

Results

Among 69 patients, the median time from biopsy to definitive surgery was 50 days (IQR 38-60), and 22 patients (31.9%) underwent surgery within 42 days. Residential distance showed a weak, non-significant association with time to surgery (β = 0.029 days/km, 95% CI −0.008 to 0.066, p = 0.118, R² = 0.036). Time to surgery did not differ significantly between distance groups (Kruskal-Wallis p = 0.25), nor did the proportion undergoing surgery within 42 days (χ²(2) = 1.24, p = 0.54).

Conclusions

No significant association was found between residential distance and time to definitive melanoma surgery within the Toowoomba regional catchment. However, most patients underwent surgery beyond 42 days, supporting further evaluation of referral pathways, service coordination and other determinants of treatment timing.

## Introduction

Australia has one of the highest melanoma incidence rates worldwide and the highest age-standardised incidence rate globally [1]. Although melanoma incidence has declined among adults aged 20-44 years and remained stable among those aged 45-64 years, mortality has fallen substantially, with reductions of 41.7% and 47.9% among men and women aged 20-44 years and 22.9% and 25.5% among men and women aged 45-64 years, respectively [2-4]. Despite these encouraging trends, timely diagnosis and treatment remain essential to optimise patient outcomes. Current guidelines recommend completion of biopsy or excision, pathology reporting, specialist referral, staging and definitive surgery within six weeks (42 days) [5]. However, delays may occur throughout the melanoma care pathway, particularly for patients in regional and rural areas who face geographic barriers to specialist services [6-8]. Cancer outcomes remain poorer in regional populations than in major cities, yet the effect of travel distance on the timeliness of melanoma surgery remains unclear [9].

Toowoomba Hospital is the principal regional referral centre for Darling Downs Health, serving a population of approximately 300,000 people across a geographically dispersed catchment of 90,000 km² [10]. Patients are referred from the Toowoomba urban area and surrounding rural and remote communities, creating potential challenges related to travel burden, specialist access and coordination across the melanoma care pathway. This study investigated whether residential distance from Toowoomba was associated with the interval between melanoma biopsy and definitive surgery among patients undergoing wide local excision with sentinel lymph node biopsy.

## Materials and methods

Study design, setting and participants

A retrospective single-centre audit was conducted at Toowoomba Hospital (TH). Consecutive patients with primary cutaneous melanoma who underwent wide local excision (WLE) with sentinel lymph node biopsy (SLNB) between 1 January 2021 and 31 December 2025 were included. Patients were eligible if both the diagnostic biopsy and definitive surgery were performed within the study period and sufficient clinical and operative data were available for analysis.

Patients were excluded if they had recurrent melanoma, underwent surgery outside Toowoomba Hospital without complete operative or pathology records, or had missing data required for the primary analysis, including the diagnostic biopsy date. Patients managed in the private sector, treated at other centres or not requiring sentinel lymph node biopsy were not included. Patients whose residential distance or time to surgery was more than three standard deviations from the cohort mean were excluded according to the prespecified outlier criterion.

Ethics approval was obtained from the relevant institutional Human Research Ethics Committee (reference number EX/2026/QTDD/127042).

Case ascertainment and data collection

Eligible patients were identified using theatre booking systems (ORMIS), pathology database searches for melanoma wide local excision and sentinel lymph node biopsy specimens, multidisciplinary team records and surgical audit logs.

Demographic, clinicopathological and operative data were extracted from electronic medical records, pathology reports and operative records. Variables collected included age, sex, residential postcode, diagnostic biopsy date, definitive surgery date and sentinel lymph node biopsy status. Residential distance from Toowoomba Hospital was estimated using Google Maps based on the patient's residential suburb and used as a proxy for geographic access to specialist care.

Exposures and outcomes

The primary exposure was residential distance from Toowoomba Hospital. The primary outcome was the interval, measured in days, between diagnostic melanoma biopsy and definitive surgical treatment with WLE and SLNB. A secondary outcome was the proportion of patients undergoing definitive surgery within the recommended 42-day timeframe.

Statistical analysis

Descriptive statistics were used to summarise patient characteristics. Continuous variables were reported as medians with interquartile ranges (IQRs), while categorical variables were reported as frequencies and percentages. Patients with missing biopsy dates were excluded from analysis. Potential outliers were defined a priori as observations lying more than three standard deviations from the cohort mean for residential distance or time to surgery and were excluded from the primary analysis.

Residential distance was analysed as both a continuous variable and a categorical variable using three predefined groups: Toowoomba urban area, <100 km from Toowoomba, and ≥100 km from Toowoomba. Patients residing within Toowoomba were assigned a nominal distance of 1 km. Differences in time to surgery between groups were assessed using the Kruskal-Wallis test, while the proportion undergoing surgery within 42 days was compared using Pearson's chi-square test. Simple linear regression was used to evaluate the association between residential distance and time to surgery. Statistical significance was defined as p<0.05 (two-sided). Analyses were performed using Microsoft Excel for Mac, version 16.110.1.

## Results

Cohort characteristics

Of 107 potentially eligible patients with primary cutaneous melanoma, 36 were excluded because the diagnostic biopsy date was unavailable, and two were excluded because residential distance or time to surgery was more than three standard deviations from the mean. Sixty-nine patients met the inclusion criteria. Baseline demographic and clinical characteristics are presented in Table 1. The cohort comprised 41 males (59.4%) and 28 females (40.6%), with a median age of 63 years (IQR 48-75). The median residential distance from Toowoomba was 83 km (IQR 1-151), and the median interval from biopsy to definitive surgery was 50 days (IQR 38-60).

**Table 1:** Baseline characteristics of the study cohort (n = 69)

Characteristic	Overall (n=69)
Age, median (IQR), years	63 (48–75)
Male sex, n (%)	41 (59.4)
Female sex, n (%)	28 (40.6)
Residential distance, median (IQR), km	83 (1–151)
Toowoomba urban area, n (%)	21 (30.4)
<100 km from Toowoomba, n (%)	20 (29.0)
≥100 km from Toowoomba, n (%)	28 (40.6)
Time to surgery, median (IQR), days	50 (38–60)
Surgery within 42 days, n (%)	22 (31.9)

The median time to surgery was 51 days (IQR 37-58) among patients residing in the Toowoomba urban area, 45.5 days (IQR 36-54.5) among those living <100 km from Toowoomba, and 51.5 days (IQR 44.5-63.5) among those residing ≥100 km from Toowoomba. Although patients residing ≥100 km from Toowoomba had the longest median interval to surgery, differences between groups were not statistically significant (Kruskal-Wallis p = 0.25) (Table 2).

Overall, 22 patients (31.9%) underwent definitive surgery within the recommended 42-day timeframe, while 47 (68.1%) underwent surgery after 42 days. The proportion treated within 42 days did not differ significantly across distance groups (Pearson's χ²(2) = 1.24, p = 0.54) (Table 2).

**Table 2:** Time to definitive surgery according to residential distance from Toowoomba P values calculated using the Kruskal–Wallis test for continuous variables and the Pearson’s Chi-square test for categorical variables. ^a^Kruskal–Wallis test ^b^Pearson’s Chi-square test

Cohort	Total	Toowoomba urban area, n = 21	Outside Toowoomba, <100 km, n = 20	≥100 km, n = 28	P value
Time to surgery (median, IQR)	50 (38 - 60)	51 (37 - 58)	45.5 (36 - 54.5)	51.5 (44.5 - 63.5)	0.25^a^
Surgery within 42 days					0.54^b^
Yes	22 (31.9%)	7 (33.3%)	8 (40.0%)	7 (25.0%)	
No	47 (68.1%)	14 (66.7%)	12 (60.0%)	21 (75.0%)	

**Figure 1:**
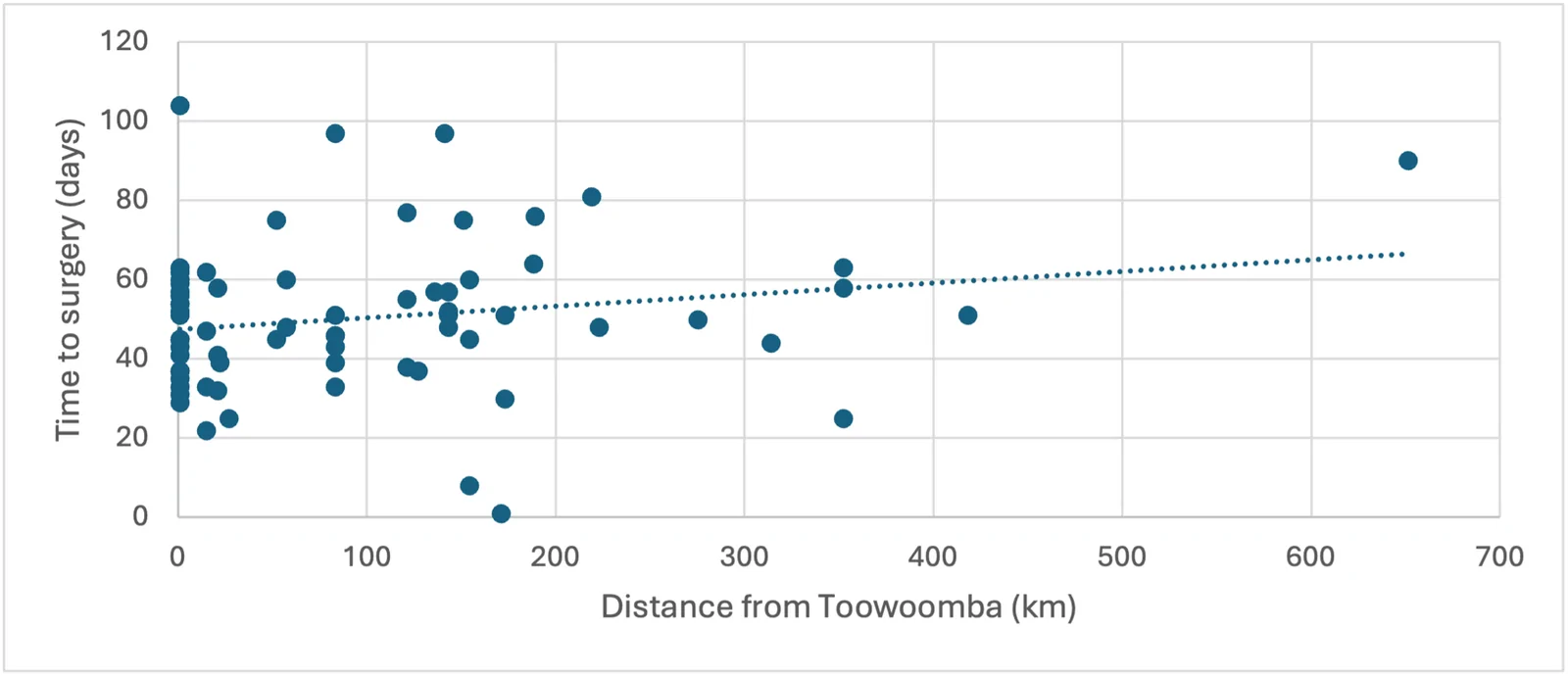
Association between residential distance from Toowoomba and time from diagnostic melanoma biopsy to definitive surgery. The dotted line represents the fitted simple linear regression line. Residential distance demonstrated a weak, non-significant positive association with time to surgery (β = 0.029 days/km, p = 0.118, R² = 0.036).

Simple linear regression demonstrated a weak positive association between residential distance and time to surgery; however, the association was not statistically significant (β = 0.029 days/km, 95% CI −0.008 to 0.066, p = 0.118, R² = 0.036; Figure 1). This corresponds to an estimated increase of approximately 2.9 days in time to surgery for every additional 100 km from Toowoomba. Residential distance explained 3.6% of the observed variation in time to surgery.

## Discussion

This audit provides a local assessment of melanoma surgical access within a regional referral service supporting a large and geographically dispersed population. Although the median interval from biopsy to surgery was 50 days and more than two-thirds of patients underwent surgery beyond 42 days, residential distance was not significantly associated with time to surgery and explained only 3.6% of the observed variation. Median surgical intervals were also broadly similar across distance groups. In a catchment spanning approximately 90,000 km², these findings suggest that patients living farther from Toowoomba did not experience substantially longer surgical intervals after entering the pathway for wide local excision and sentinel lymph node biopsy [10].

The findings therefore present a mixed picture. The regional referral model may support broadly comparable access across geographic groups; however, overall timeliness remains an area for further evaluation. The absence of an association with distance does not explain why most patients underwent surgery beyond 42 days. Melanoma surgery with sentinel lymph node biopsy requires coordination between referring clinicians, pathology, specialist surgical assessment, nuclear medicine and operating theatre services. Delays may therefore arise at several stages, but the present study measured only the total biopsy-to-surgery interval and could not determine where they occurred.

A pathway-based audit would help identify opportunities for improvement by separately examining the intervals from biopsy to pathology reporting, pathology to referral, referral to specialist assessment, and surgical assessment to operation. Recording reasons for delay could determine whether future initiatives should focus on referral completeness, outpatient access, coordination between nuclear medicine and theatre services, or patient-level barriers [11]. Improved capture of externally performed biopsy dates would also strengthen future evaluation of regional access.

This study was limited by its retrospective single-centre design, relatively small and selected cohort, and missing biopsy dates. The study cohort represents a selected subgroup of patients undergoing wide local excision with sentinel lymph node biopsy at Toowoomba Hospital rather than all patients diagnosed with melanoma within the Darling Downs region. Furthermore, restriction to patients undergoing sentinel lymph node biopsy may limit generalisability to patients with very early or metastatic disease [12]. Postcode-based distance may not reflect actual travel time, transport access or socioeconomic disadvantage, while unmeasured clinicopathological and service factors may also have influenced treatment timing [13-15]. Nevertheless, this study establishes a useful baseline for evaluating melanoma surgical care across the Darling Downs and provides a framework for identifying where future pathway improvements should be directed.

## Conclusions

In this regional referral cohort, residential distance was not significantly associated with time from melanoma biopsy to definitive surgery and explained only a small proportion of the observed variation in treatment timing. Nevertheless, the median surgical interval exceeded the recommended 42-day timeframe, with more than two-thirds of patients undergoing surgery beyond this benchmark. These findings suggest that geographic distance alone is unlikely to account for delays within the Toowoomba melanoma pathway. Future pathway-based studies should examine referral timing, diagnostic processing, specialist assessment, theatre coordination and patient-level barriers to identify modifiable contributors and support targeted improvements in regional melanoma care.
